# Iron limitation induces motility in uropathogenic *E. coli* CFT073 partially through action of LpdA

**DOI:** 10.1128/mbio.01048-24

**Published:** 2024-06-14

**Authors:** A. E. Frick-Cheng, A. E. Shea, J. R. Roberts, S. N. Smith, M. D. Ohi, H. L. T. Mobley

**Affiliations:** 1 Life Sciences Institute, University of Michigan, Ann Arbor, Michigan, USA; 2 Department of Microbiology and Immunology, University of South Alabama Medical School, Mobile, Alabama, USA; 3 Department of Anesthesiology, University of Michigan Medical School, Ann Arbor, Michigan, USA; 4 Department of Microbiology and Immunology, University of Michigan Medical School, Ann Arbor, Michigan, USA; Mississippi State University, Mississippi State, Mississippi, USA

**Keywords:** flagellar motility, iron regulation, urinary tract infection, flagellar gene regulation, pathogenesis

## Abstract

More than half of women will experience a urinary tract infection (UTI) with most cases caused by uropathogenic *Escherichia coli* (UPEC). Bacterial swimming motility enhances UPEC pathogenicity, resulting in more severe disease outcomes including kidney infection. Surprisingly, the connection between motility and iron limitation is mostly unexplored despite the lack of free iron available in the host. We sought to investigate a potential connection between iron restriction and regulation of motility in UPEC. We cultured *E. coli* CFT073, a prototypical UPEC strain, under iron limitation and observed that CFT073 had elevated *fliC* (flagella) promoter activity, and this iron-specific response was repressed by the addition of exogenous iron. We confirmed increased flagellar expression in CFT073 by measuring *fliC* transcript, FliC protein, and surface-expressed flagella under iron-limited conditions. Interestingly, known motility regulator *flhDC* did not have altered transcription under these conditions. To define the regulatory mechanism of this response, we constructed single knockouts of eight master regulators and found the iron-regulated response was lost in *crp, arcA,* and *fis* mutants. Thus, we focused on the five genes regulated by all three regulators. Of the five genes knocked out, the iron-regulated motility response was most strongly dysregulated in the *lpdA* mutant, which also resulted in significantly lowered fitness in the murine model of ascending UTI, both against the WT and a non-motile *fliC* mutant. Collectively, we demonstrated that iron-mediated motility in CFT073 is partially regulated by *lpdA*, which contributes to the understanding of how uropathogens differentially regulate motility mechanisms in the iron-restricted host.

**IMPORTANCE:**

Urinary tract infections (UTIs) are ubiquitous and responsible for over five billion dollars in associated health care costs annually. Both iron acquisition and motility are highly studied virulence factors associated with uropathogenic *Escherichia coli* (UPEC), the main causative agent of uncomplicated UTI. This work is innovative by providing mechanistic insight into the synergistic relationship between these two critical virulence properties. Here, we demonstrate that iron limitation has pleiotropic effects with consequences that extend beyond metabolism and impact other virulence mechanisms. Indeed, targeting iron acquisition as a therapy may lead to an undesirable enhancement of UPEC pathogenesis through increased motility. It is vital to understand the full breadth of UPEC pathogenesis to adequately respond to this common infection, especially with the increase of antibiotic-resistant pathogens.

## INTRODUCTION

Urinary tract infections (UTIs) are a prominent health concern worldwide; they are the second most frequent infectious disease and disproportionately affect women, children, the elderly, immune-compromised, and hospitalized populations (1–3). Most uncomplicated UTIs are caused by uropathogenic *Escherichia coli* (UPEC) and are restricted to the bladder (4, 5). However, if left untreated, these infections can ascend to the kidneys causing permanent tissue damage and renal scarring; 80% of cases of pyelonephritis are caused by UPEC (6–8). In extreme cases, bacteria can spread to the bloodstream potentially progressing to sepsis (8, 9); about 25% of sepsis cases originate from UTIs (10). Even in the less severe, non-ascending cases of UTI, the financial burden and decreased quality of life are damaging to these patients (3, 11).

An essential and well-studied mechanism of bacterial dissemination from the bladder to the kidneys is motility (1, 12). UPEC has been detected in murine kidneys as early as 6 hours post inoculation (hpi) into the bladder (12). Flagella, expressed on the bacterial surface, propel the bacteria in a targeted direction utilizing chemotactic mechanisms (13, 14). Many studies have been conducted in prototype commensal strains of *E. coli* to understand the mechanical function and regulation of flagella and the chemoattractant stimuli that facilitate directed movement (15, 16). However, the established dogma of flagellar movement and regulation from non-pathogenic strains may differ in pathogenic varieties.

Strains of *E. coli* that cause human disease are genetically diverse from non-pathogenic serotypes (17, 18). For example, Trg and Tap chemoreceptors, which direct bacteria toward ribose, galactose, and dipeptides (19, 20), are less prevalent and functional among UPEC isolates than fecal or commensal strains and even more so when compared to diarrheal strains (21). Because the genetic and phenotypic display of these pathogens are varied, it is likely that regulatory mechanisms of key processes that include bacterial motility are as well. Another example of divergence between pathogenic and commensal *E. coli* strains is the prevalence of iron importation systems. Uropathogens can import iron using multiple siderophore systems not found in commensal isolates (22, 23). Iron acquisition is especially important for pathogens as the human host is an iron-restricted environment (24). The ferric uptake regulator, Fur, was found to have additional binding sites within the pathogenicity islands of UPEC compared to commensal strains, including many of these additional iron uptake systems, while transcription of the core genome remained consistent (25).

We observed an increased motility phenotype in uropathogenic strain *E. coli* CFT073 under chemically induced iron-limited conditions. This phenotype corresponded with an increase in flagellin transcript, protein, and surface expression of flagella. Knowing that the well-studied Fur regulator was likely not facilitating this regulation in our tested conditions (25, 26), we took a forward genetic screening approach to identify the mediator of the iron-restricted enhanced motility phenotype. Through systematic deletion of regulatory genes, we ultimately found that deletion of the gene encoding dihydrolipoyl dehydrogenase, *lpdA*, led to a loss of iron-mediated motility as well as fitness *in vivo*. Interestingly, this gene had not been previously linked to either iron or motility. Collectively, we demonstrated that the deletion of a highly conserved metabolism-related gene alters swimming motility and leads to a defect in the uropathogenesis both *in vitro* and *in vivo*.

## RESULTS

### The flagellar promoter is highly active under iron-restricted conditions

**Fig 1:**
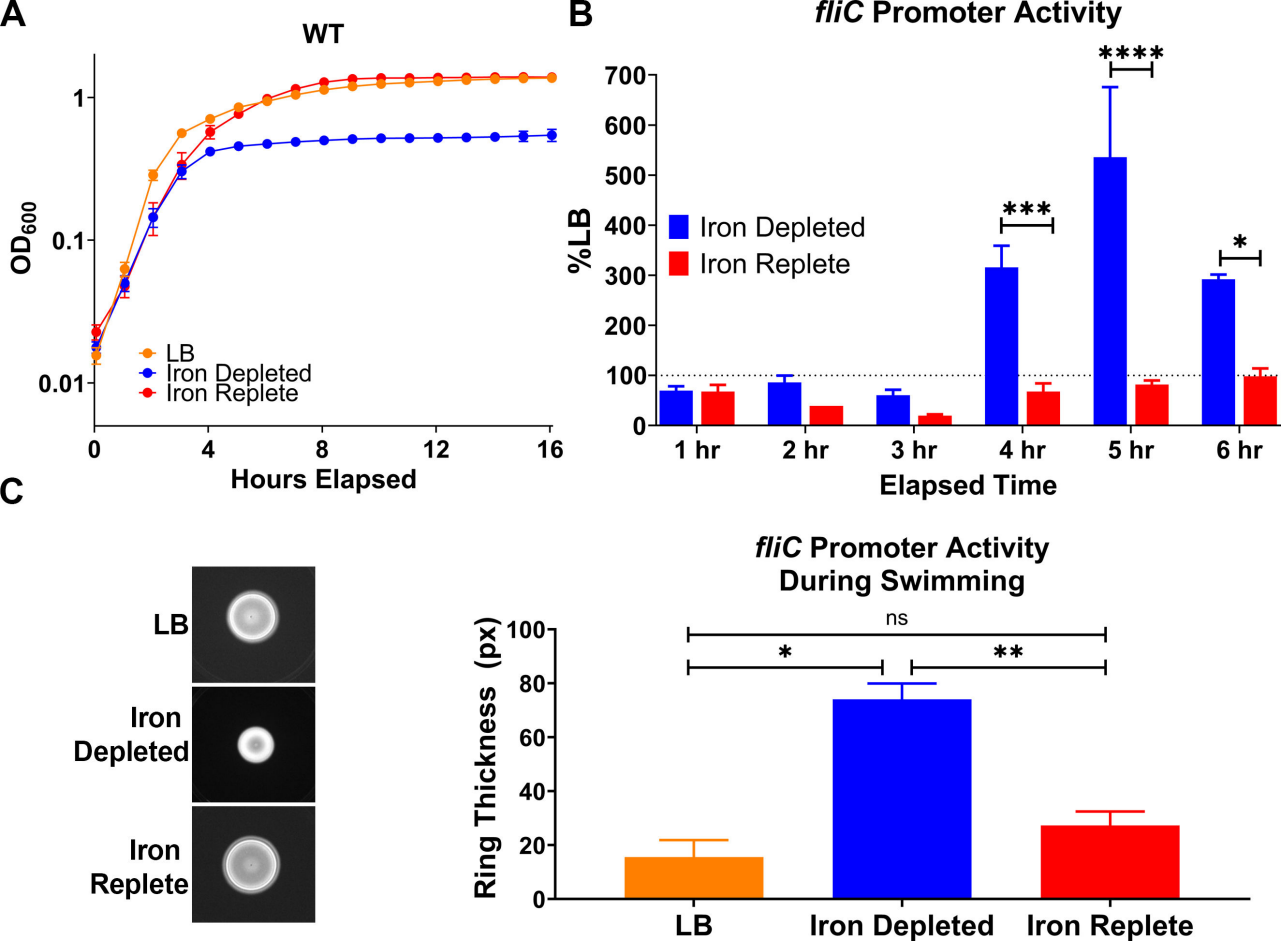
*fliC* promoter activity is elevated under iron-depleted conditions. (**A**) Uropathogenic *E. coli* CFT073 was cultured in LB, iron-depleted LB (supplemented with 300 µM 2,2 dipyridyl, an iron chelator) and iron-replete LB (supplemented with 300 µM dip and 300 µM FeCl_3_) 16 hours with aeration. Growth curves display averages from four biological replicates; error bars indicate ±SEM. (**B**) *fliC* promoter activity was assayed with a luciferase reporter over 6 hours in iron-depleted and iron-replete conditions and normalized to LB medium. Bars represent the mean from five biological replicates; error bars are ±SEM. Asterisks compare iron-depleted and iron-replete conditions, using two-way ANOVA with two-stage linear procedure of Benjamini Krieger and Yekutieli for multiple test corrections: *P < 0.05, ***P < 0.001, ****P < 0.0001. (**C**) *fliC* promoter activity was assessed during motility for 16 hours in semisoft agar at 37°C in LB, iron-depleted, and iron-replete conditions. Representative image shows luciferase activity, and the thickness of the outer ring was quantified in a bar graph. Bars display averages from four biological replicates; error bars indicate ±SEM. Asterisks compare iron-depleted and iron-replete LB medium, using two-way ANOVA Tukey’s multiple test corrections: *P < 0.05, **P < 0.01.

RNAseq performed on a siderophore-deficient mutant of clinical UPEC isolate HM7 revealed that flagella-related genes were significantly upregulated when the mutant was cultured in M9 minimal medium (27) (Table S1). We reasoned that this phenomenon may be occurring due to iron restriction and wanted to determine if this was also relevant in the highly characterized UPEC type strain CFT073 (17). However, unlike HM7, CFT073 has three siderophores (28) and would necessitate creating a triple mutant to re-create similar experimental conditions. Therefore, we decided to take a chemical approach and test conditions in an iron-depleted medium. First, we determined the concentration of iron chelator 2,2 dipyridyl (dip) in LB medium that would restrict iron availability but not substantially affect bacterial growth and survival (Fig. S1). We observed that 300 µM dip would serve as the ideal concentration for minimal growth inhibition, and growth could be rescued with the addition of exogenous 300 µM FeCl_3_ (Fig. 1A). These conditions were utilized throughout this study.

Motility is an important process required for the ascension of UPEC into the upper urinary tract (1, 12). Lane et al. (12) documented the kinetics and necessity of flagellar expression during active murine UTI, utilizing a luciferase-expressing vector under the control of the native *fliC* promoter (P*_fliC_*-lux). We used this vector to measure the activity and kinetics of the flagella subunit promoter under iron-depleted and iron-replete conditions, relative to unaltered LB medium. There were significant increases in *fliC* promoter activity at 4, 5, and 6 hpi (Fig. 1B). The largest difference between the iron-depleted and replete conditions was at 5 hpi, where there was over a fivefold increase in *fliC* promoter activation coupled with repression down to 81% of original levels in the iron-replete condition (Fig. 1B). Interestingly, the relatively slow response of the *fliC* promoter implies that this response is not mediated by the classical iron-regulator, Fur, which can control responses as quick as 15 minutes from stimulus (29).

Because promoter activity is the first step in the central dogma of molecular biology, we wanted to assess if there was a commensurate increase in the functional activity of the flagella. To do so, we used the *fliC* reporter construct to visualize the population of bacteria with high *fliC* promoter activity during swimming motility. Lane et al. (12) showed that the leading edge of the swimming colony has high *fliC* promoter activity, which was visualized using the P*_fliC_*-lux construct. Therefore, we measured the thickness of the luminescence of the leading edge of swimming bacteria in LB, iron-depleted, and iron-replete swim agar (Fig. 1C; Fig. S2). While the overall swim diameter (measured at 16 hours) in the iron-depleted condition was smaller when compared to LB, the *fliC* leading edge was four times thicker, indicating a stronger *fliC* promoter activity (Fig. 1C). This trend was repressed back to similar levels observed in the LB control by the addition of 300 µM FeCl_3_ exogenous iron in the presence of the iron chelator (Fig. 1C). These data demonstrate that CFT073 upregulates flagella under iron-limited conditions.

### Flagella are upregulated at the gene, protein, and structural level

**Fig 2:**
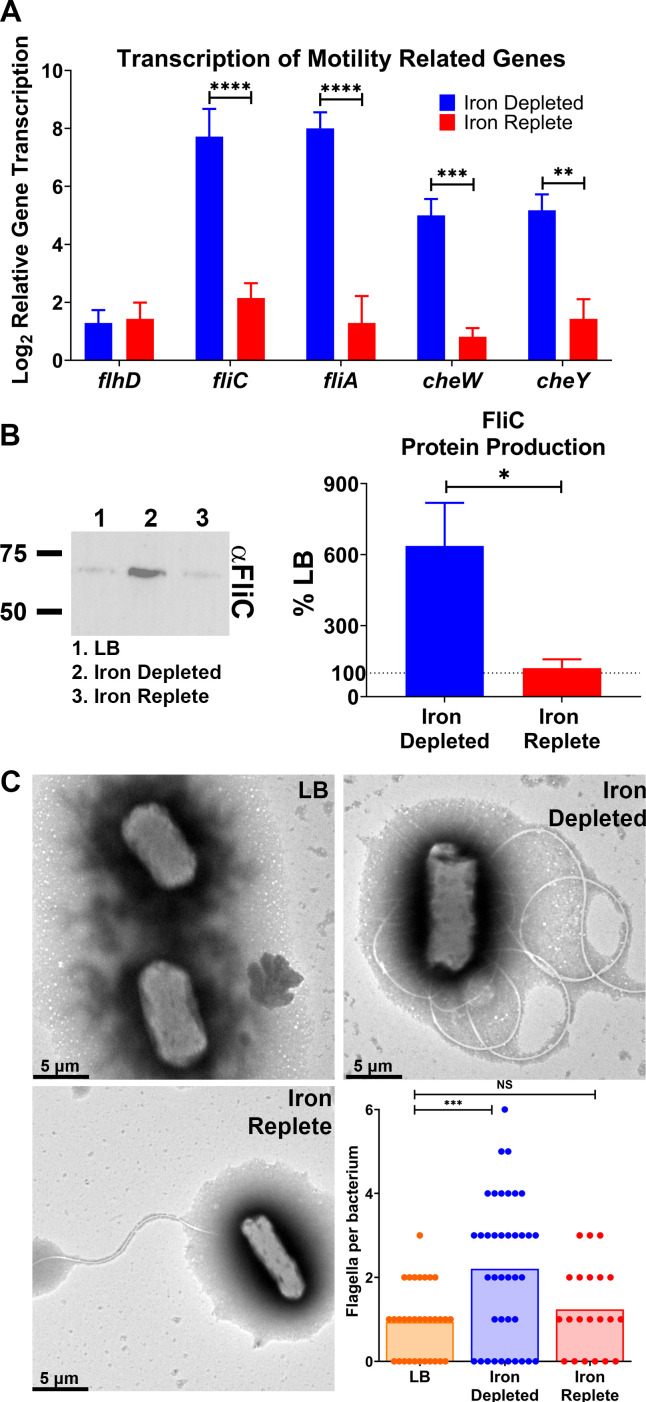
*fliC* gene transcription and protein abundance are elevated under iron limitation, leading to an increase in surface-expressed flagella. CFT073 was cultured in LB, iron-depleted LB (LB supplemented with 300 µM dip), and iron-replete LB (LB supplemented with 300 µM dip and 300 µM FeCl_3_) for 5 hours. (**A**) RNA was extracted, and transcription of indicated genes was assayed via qRT-PCR and compared to the LB control. *n* = 4. (**B**) Cultures were normalized by OD_600_, and the whole-cell lysate was immunoblotted. Densitometry of FliC was calculated, *n* = 6, and representative immunoblot is shown. Bars indicate mean; error bars are ±SEM, *, *P* < 0.05, ****P < 0.0001 determined by paired *t*-test. (**C**) Representative electron micrographs of CFT073 cultured in either LB, iron-depleted, or iron-replete conditions. Bacteria were fixed with 2.5% glutaraldehyde and stained with 1% phosphotungstic acid and imaged via TEM. The flagella per cell were quantified; each dot represents an individual bacterium. Bars represent the mean, ***P < 0.001 determined by ordinary one-way ANOVA with Dunnett’s multiple comparisons test. NS, not significant.

To delve further into this motility response, we chose a panel of motility-associated genes and examined their transcription during iron-depleted and -replete growth conditions. Chemotaxis genes (*cheW* and *cheY*) and flagella machinery genes (*fliC* and *fliA*) had increased transcription under iron-depleted conditions, but interestingly, master regulator *flhDC* (30, 31) did not (Fig. 2A). The magnitude of upregulation was the greatest in the flagellar genes, and transcription of all genes was repressed when iron was added back to cultures (Fig. 2A). These trends were also observed at a protein level; CFT073 grown in iron-deprived conditions had a sixfold increase in FliC protein (Fig. 2B). Congruently, an increase in the average number of flagella per bacterial cell was observed under iron limitation and quantified via electron microscopy (Fig. 2C). A single flagellum decorated the bacterium in LB or iron-replete conditions, while this number increased to two under iron depletion (Fig. 2C). These data, in conjunction with those in Fig. 1, prove that the iron responsiveness of the flagella machinery in UPEC extends from promoter activity to the production of flagellar structures.

### Loss of global regulators *fis*, *crp*, and *arcA* dysregulates the iron-mediated motility response

We wanted to understand what is transcriptionally driving the iron-mediated motility response. FlhDC is a well-known motility master regulator (30), while Fur is a highly studied iron-responsive transcription factor (32). While a study has shown that Fur weakly binds the FlhD promoter (33), the effect we are observing is FlhD independent (Fig. 2A). Furthermore, that same study (33) showed Fur does not directly bind the *fliC* promoter, a result corroborated by several other studies (25, 26). We also analyzed the upstream intergenic region of *fliC* using the bioinformatics tool BPROM (Softberry.com), which did not uncover any putative fur boxes. Altogether, this evidence suggests that Fur is not a direct regulator of *fliC*.

**Fig 3:**
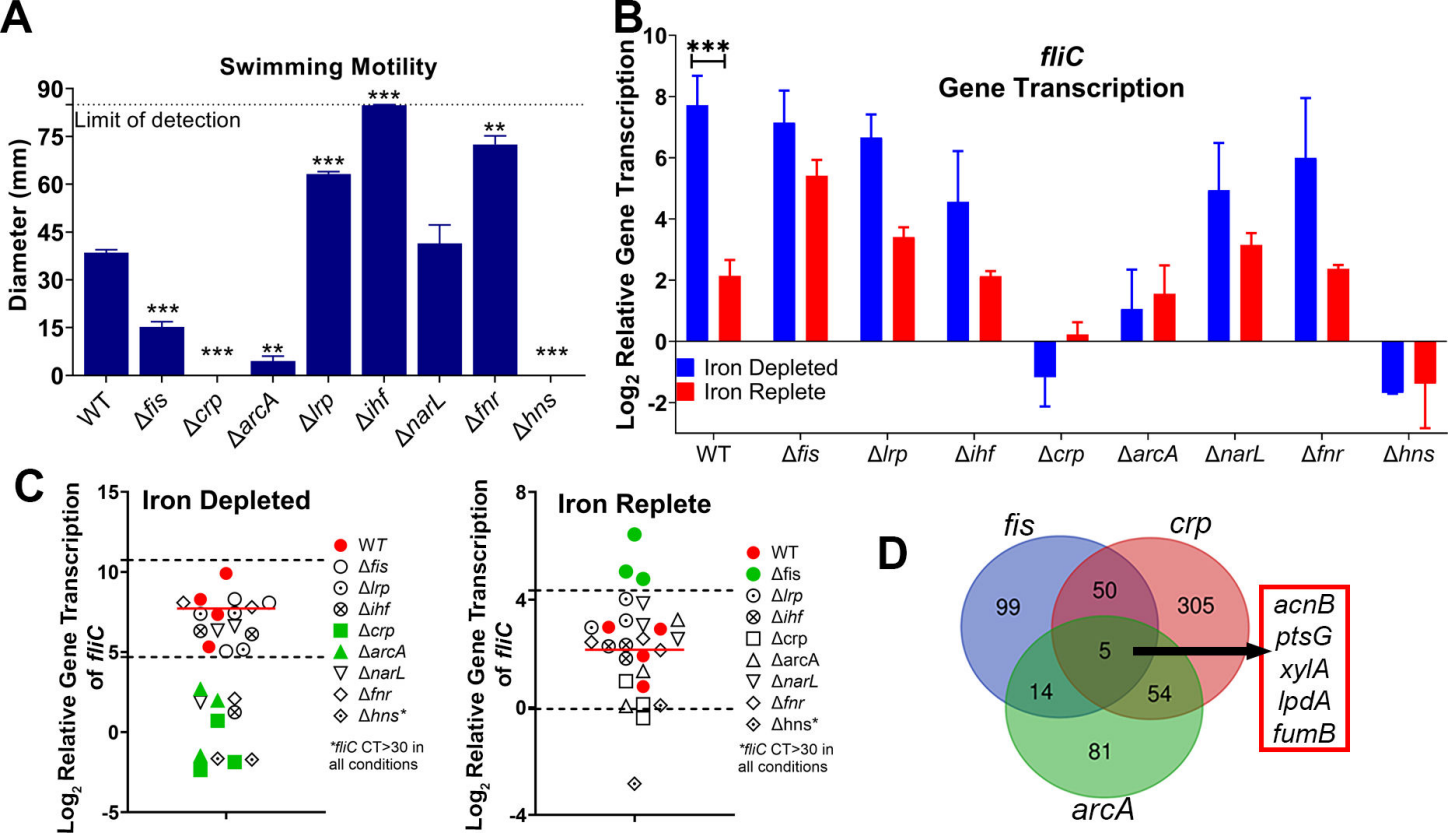
Loss of *fis, crp,* or *arcA* disrupts iron-mediated *fliC* transcription. Master regulator mutants were generated using λ-red mutagenesis. Strains were cultured for 5 hours in LB, iron-depleted (LB supplemented with 300 µM dip), and iron-replete (LB supplemented with 300 µM dip and 300 µM FeCl_3_). (**A**) Motility was assessed in semisoft tryptone agar at 30°C, *n* = 4. **P < 0.01, ***P < 0.001, repeated measures ANOVA (RM ANOVA), Dunnett’s correction. (**B**) *fliC* transcription was assessed and normalized to LB as in previous assays. *n* = 4, bars indicate mean; error bars are ±SEM, ***P < 0.001 determined by two-way ANOVA with Sidak’s multiple comparison test. (**C**) Strategy to cull transcription factor hits. (**D**) Genes regulated by *fis, crp,* and *arcA*.

Thus, we decided to take a top-down approach and generated deletions in eight master transcriptional regulators (34), which have the largest downstream regulatory influence, incorporating the largest number of transcription factors as possible. None of these mutants exhibited drastically different growth under iron-depleted and -replete conditions (Fig. S3). We examined the swimming ability of these mutants to select candidates for follow-up (Fig. 3A). The Δ*fis,* Δ*crp,* Δ*arcA,* and Δ*hns* mutants either had reduced motility or were non-motile, while the Δ*lrp,* Δ*ihf*, and Δ*fnr* mutants were hyper-motile (Fig. 3A). Then, we assessed the loss of iron-regulated flagellin expression in these mutants by comparing the transcription of *fliC* in iron-depleted and -replete medium (Fig. 3B). If all biological replicates of a mutant were over two standard deviations different from the mean WT value, we marked it as a gene of interest (Fig. 3C). Based on these criteria, Δ*fis,* Δ*crp*, Δ*arcA*, and Δ*hns* lost the ability to regulate *fliC* in an iron-dependent manner (Fig. 3B and C). However, Δ*hns* had nearly undetectable levels *of fliC* via qRT-PCR (CT value >30) in any condition and was therefore eliminated from further study. We examined the direct regulons of Δ*fis,* Δ*crp*, and Δ*arcA* to narrow down the potential iron-mediated motility mechanism in UPEC (34) and found five genes were present in all three regulons (Fig. 3D). These were *acnB*, *ptsG*, *xylA*, *lpdA*, and *fumB*. Four of these genes are involved in metabolism-related processes, and three of them have metallic cation-binding sites (*fumB*, *acnB*, and *xylA*).

### Deletion of *lpdA,* encoding lipoamide dehydrogenase, causes loss of iron-regulated *fliC* transcription and motility

**Fig 4:**
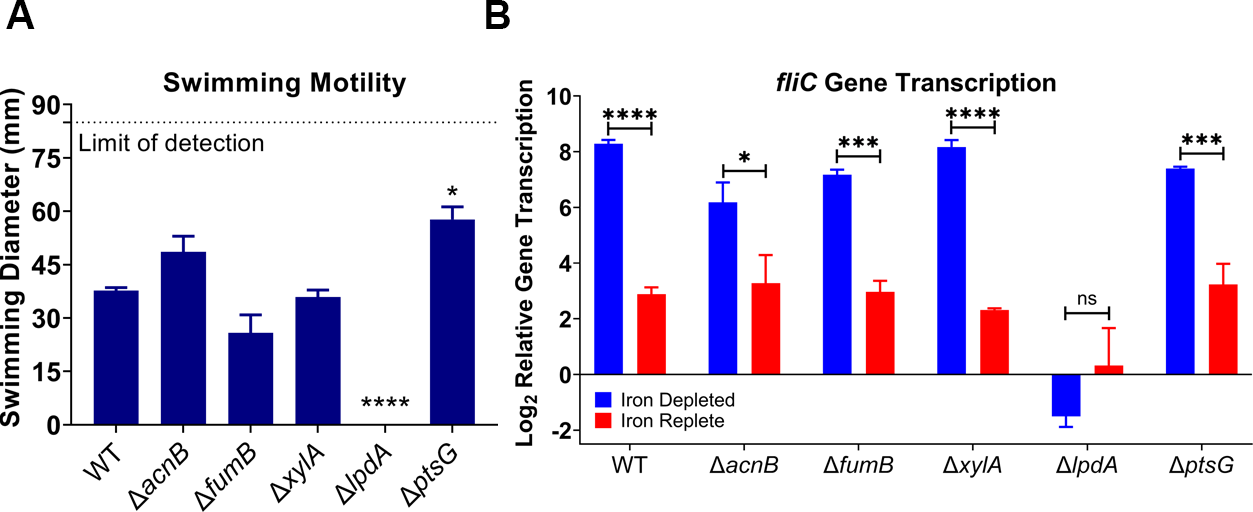
Loss of *lpdA* disrupts iron-mediated *fliC* response. Mutants were generated using λ-red mutagenesis. Strains were cultured for 5 hours in LB, iron-depleted LB (LB supplemented with 300 µM dip), and iron-replete LB (LB supplemented with 300 µM dip and 300 µM FeCl_3_). (**A**) Motility was assessed in semisoft tryptone agar at 30°C, *n* = 6, *P < 0.05, ****P < 0.0001, RM ANOVA, Dunnett’s correction. (**B**) *fliC* transcription levels were assessed 5 hours post inoculation and normalized to LB as in previous assays. *n* = 4, bars represent the mean; error bars are ±SEM, *P < 0.05, ***P < 0.001, ****P < 0.0001 determined by two-way ANOVA with Sidak’s multiple comparison test.

To further pursue the regulatory mechanism of iron-mediated motility, we generated knockouts in each of the previously determined genes of interest and examined the swimming motility of these mutants (Fig. 4A) and transcription of *fliC* (Fig. 4B). The Δ*lpdA* mutant was the only strain that was non-motile in swim agar (Fig. 4A) and where *fliC* transcription was no longer regulated by iron (Fig. 4B). Even with these two defects, the mutant was able to grow similarly to WT (Fig. S4). LpdA is the E3 component of the pyruvate dehydrogenase complex, which converts pyruvate into acetyl-CoA which feeds into the TCA cycle, connecting it with glycolysis under aerobic conditions (35, 36). LpdA binds the coenzyme FAD, which facilitates the oxidation of dihydrolipoate back to lipoate, producing FADH_2_ in the process (35, 36). Loss of LpdA results in accumulation of intracellular pyruvate and glucose (35). However, *E. coli* is still able to use glucose as a sole carbon source in the absence of LpdA due to another enzyme, PoxB, that can directly convert pyruvate into CO_2_ and acetate (35).

**Fig 5:**
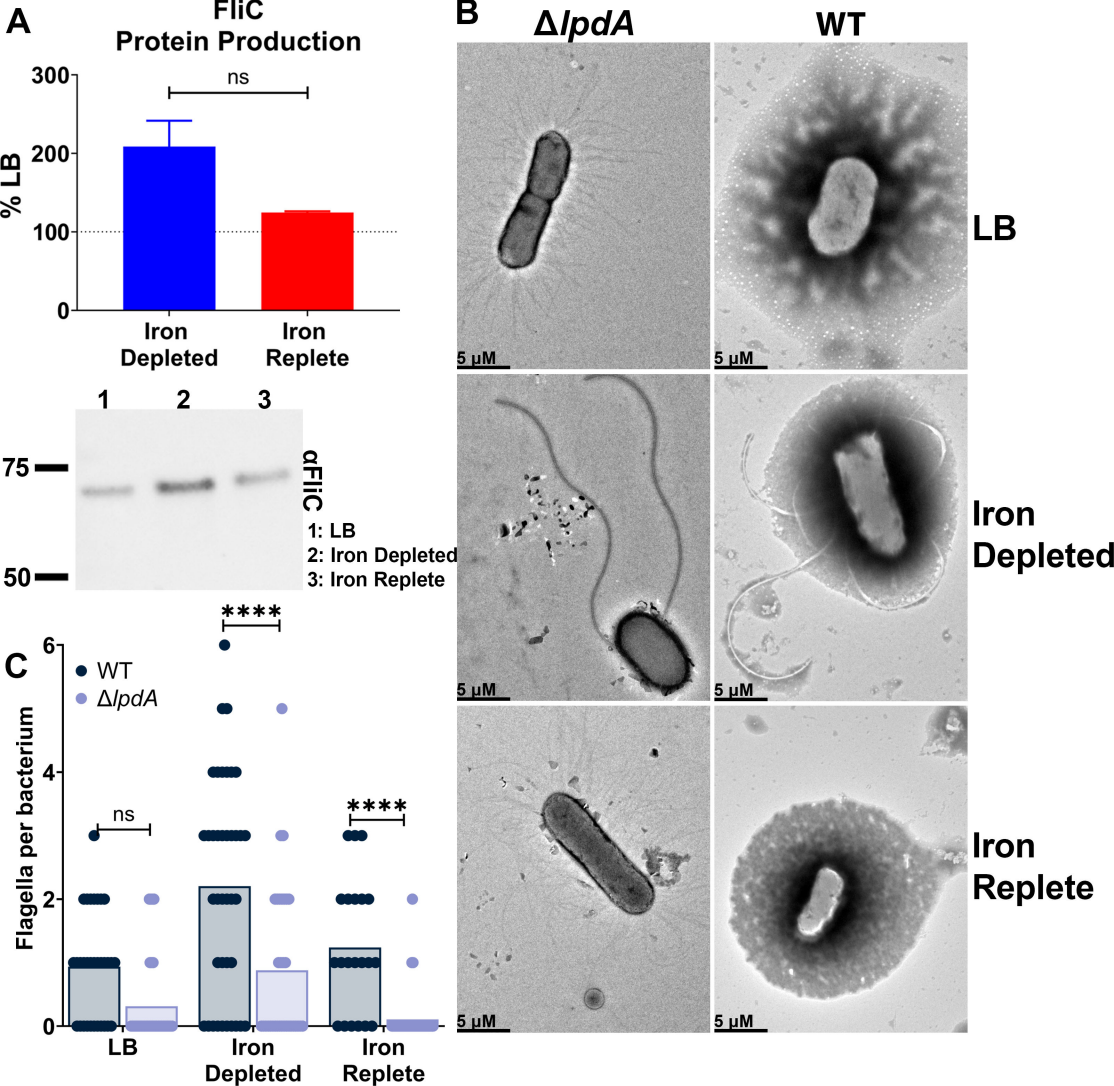
Loss of *lpdA* attenuates FliC protein production and flagella production under iron limitation, resulting in host fitness defects. (**A**) Cultures were normalized by OD_600_, and the whole-cell lysate was immunoblotted. Densitometry of FliC was calculated, *n* = 4, and representative immunoblot is shown. Bars represent the mean; error bars are ±SEM, not significant (ns) determined by unpaired *t*-test. (**B**) Representative electron micrographs of CFT073 cultured in either LB, iron-depleted, or iron-replete conditions. Bacteria were fixed with 2.5% glutaraldehyde and stained with 1% phosphotungstic acid and imaged via TEM. (**C**) The number of flagella per bacterium was quantified; each dot represents an individual bacterium, and the bar represents the mean. Asterisks compare WT and *lpdA* mutant in LB, iron-depleted, or iron-replete conditions using two-way ANOVA with Sidak’s multiple comparisons test, ****P < 0.0001.

Knowing that gene transcription of *fliC* was unresponsive in the *lpdA* mutant (Fig. 4B), we turned our attention to potential deficiencies at the protein and structural assembly levels. Δ*lpdA* produced about twofold more FliC protein in iron-depleted conditions compared to iron-replete conditions (Fig. 5A); however, this change was not significant. The magnitude was less compared to the WT, suggesting that the loss of *lpdA* partially, but not completely, attenuates the response. We continued to see this partial attenuation when quantifying flagella per bacterium in the *lpdA* mutant. There was a mild increase of flagellar production under iron limitation (Fig. S5), but the magnitude of this effect was significantly smaller when compared to the effect observed with WT cells (Fig. 5B and C). These results reflect the changes seen via western blot and indicate a general dysregulation of flagellar production, though it indicates *lpdA* is likely one of several factors controlling the iron-mediated regulation of flagella rather than the sole contributor.

### Surface-expressed flagella are reduced in the *lpdA* mutant, but their expression can be genetically complemented

We have confirmed loss of *lpdA* partially dampens iron-mediated flagellar upregulation (Fig. 5). To confirm these phenomena were a direct result of *lpdA*, we complemented the mutant in *trans* under the control of its native promoter. The swimming motility of the non-motile mutant was restored to WT levels with the addition of the *lpdA* vector compared to the empty vector negative control (WT eV) (Fig. S6A). We were also able to rescue flagella production in both the LB and iron-deplete conditions in the genetically complemented strain (Δ*lpdA^+lpdA^*) (Fig. S6B and C). In iron-replete conditions, there was no significant difference between the *lpdA* mutant expressing empty vector and WT eV, and Δ*lpdA^+lpdA^*, though the results were trending toward significance (Fig. S6D). However, flagella production of the WT eV was also altered, which may account for this result. Ultimately, using a systematic approach, we determined that loss of *lpdA* causes the iron-dependent swimming motility responsiveness to be muted, with an overall dysregulation of flagella at the genetic level.

### *lpdA* contributes to uropathogenesis through metabolic and motility dysfunction

**Fig 6:**
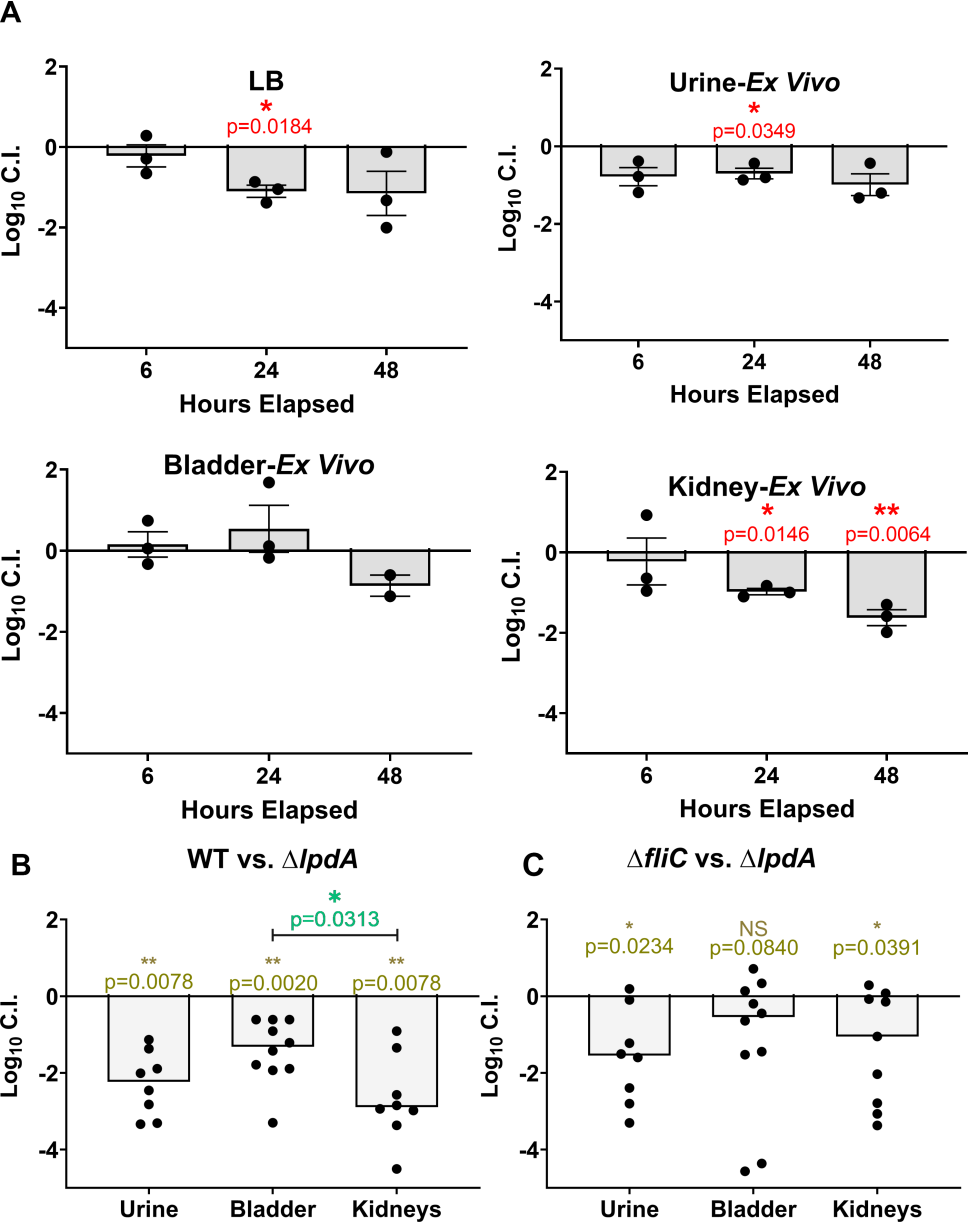
Loss of *lpdA* results in fitness defects both *ex vivo* and *in vivo*. (**A**) WT CFT073 and *lpdA* mutant were combined in a 1:1 ratio and cultured in either LB, human urine, homogenized naïve murine bladder, or kidney. CFU of either WT or mutant was determined 6, 24, and 48 hours post inoculation. Dots are biological replicates, bars represent mean, and error bars are ±SEM. Significance was determined using one sample *t*-test with indicated *P* values. (**B**) WT CFT073 and the *lpdA* mutant (**C**) *fliC* and *lpdA* mutant were combined in a 1:1 ratio and transurethrally inoculated into the bladders of CBA/J mice with organs and urine collected 48 hours post infection. Bars represent the median, while each dot represents an individual animal, *n* = 10. Significance was determined using Wilcoxon’s signed-rank test with indicated *P* values, and burden between bladder and kidneys was compared using Wilcoxon’s test.

Finally, we wanted to assess the effect of *lpdA* on uropathogenesis. Since the *lpdA m*utant has potential metabolic dysregulation, we performed a co-challenge between the mutant and WT in LB, human urine, and naïve murine organ homogenates from bladders and kidneys. Growing bacteria in *ex vivo* organ homogenates has been shown to be an excellent proxy of their metabolic needs *in vivo* (37). Each condition was inoculated with an equal amount of WT and *lpdA* mutant and cultured statically at 37°C with timepoints taken at 6, 24, and 48 hpi to assess the fitness of the mutant. We found the *lpdA* mutant had no defect when grown in bladder homogenates and only had mild defects in LB and urine 24 hpi that resolved 48 hpi (Fig. 6A). The strongest defect was in the kidneys with an ~10-fold defect at 24 hpi, which increased to ~40-fold by 48 hpi (Fig. 6A).

To understand the full impact that *lpdA* might have on virulence, we transurethrally inoculated female CBA/J mice with equal ratios of WT and *lpdA* mutant using the murine model of ascending UTI and allowed the infection to progress for 48 hours (38). Loss of *lpdA* resulted in ~150-fold defect in the urine, over a 20-fold fitness defect in the bladder, and ~750-fold defect in the kidneys (Fig. 6B). These results match the trends observed in the *ex vivo* experiments, but given the larger defect magnitude *in vivo* (Fig. S7A), there are potentially metabolic-independent effects that account for this result. The primary candidate for this result would be the loss of iron-mediated motility, especially given that the defect was the strongest in the kidneys, an organ site where swimming motility is vital (12, 39).

To fully delineate the contribution of metabolic dysregulation and any other effects that would occur in the *lpdA* mutant, we generated a *fliC* mutant and performed an *in vivo* co-challenge between the two strains (Fig. 6C). Like the *lpdA* mutant, the *fliC* mutant is non-motile; therefore, the difference between the two strains would be based on genetic and metabolic dysregulation. There was no defect of the *lpdA* mutant in the bladder, but there was a mild defect in the urine (~30-fold) and kidneys (~10-fold). This indicates that while metabolic dysfunction does contribute, the loss of motility is the main driver of the substantial loss of fitness with the *lpdA* mutant compared to WT *in vivo* (Fig. S7B). These results show the importance of *lpdA* and the iron-regulated motility for the full pathogenesis of UPEC.

## DISCUSSION

Most iron within the human host is sequestered by specific host proteins to control bacterial growth (24). In response, pathogenic bacteria employ numerous well-studied strategies to acquire iron from these sequestered sources (40), and UPEC, the main causative agent of uncomplicated UTI, is no exception. Indeed, iron acquisition is so crucial to pathogenesis that antigens from these systems have been used in trial vaccines against UPEC (41, 42).

Swimming motility is another highly studied arm of UPEC virulence; it enables bacteria to ascend to the kidneys more effectively and rapidly (12). Despite the significance of these two virulence traits, to date, there are limited studies linking the regulation of these two virulence mechanisms in UPEC. Here, we demonstrate that an iron-restricted environment leads to an increase in motility-related functions, spanning the regulatory dogma, from gene transcription of flagellar components to an increase of flagella on the surface of the bacteria.

Under iron limitation, uropathogenic type strain *E. coli* CFT073 had significant increases in *fliC* promoter activity, transcript, and protein production (Fig. 1B; Fig. 2A and B). This response extended to functional consequences; CFT073 cultured under iron-depleted conditions was decorated with more flagella (Fig. 2C), and the leading edge of swimming bacteria expressed *fliC* more strongly (Fig. 1C). The addition of exogenous iron was able to re-repress all responses (Fig. 1 and 2), demonstrating that these are iron-specific responses and not attributable to off-target effects of the iron chelator. Perhaps an iron-limited environment could serve as one of many cues to the bacteria upon entering the host to upregulate flagellar machinery.

It is interesting to speculate that iron-limited motility in *E. coli* has remained understudied because the seminal papers used commensal isolate K12, and conclusions drawn from a non-pathogenic isolate are not always applicable to a pathogenic strain. For example, while the motility of K12 is inhibited at 37°C (15, 43), our study shows robust *fliC* transcription and upregulation in UPEC strain CFT073 at this temperature (Fig. 1C and 2A) as well as an ability to swim (Fig. 1C). Furthermore, while our study reveals a strong, reproducible response where iron limitation induces *fliC* transcription in CFT073, the opposite has been shown in K12; increasing iron concentration elevated *fliC* transcription in a K12 derivative (44). This starkly emphasizes the differential regulatory responses between commensal and pathogenic isolates. Perhaps the iron-limited motility response could be used as a distinguishing feature of uropathogens, or the response could be characteristic of pathogenic *E. coli* that infect other body sites, such as the bloodstream or gut. Determining the conservation of this response mechanism among other uropathogens is a future research direction.

After establishing the consistency of the iron-regulated motility response, our next objective was to define the underlying mechanism. Two widely studied iron regulators are Fur (32, 45) and RyhB (a small RNA) (46). However, a recent paper (25) defined both the direct and indirect regulons of Fur and RyhB in CFT073, and not a single component of the flagellar machinery was uncovered. Though, interestingly, another study found that Fur weakly binds to the FlhD promoter (33). Given the effect was FlhD independent, we decided to investigate eight master regulators (Fig. 3). Loss of *fis, crp,* or *arcA* resulted in dysregulation of *fliC* gene transcription in iron-depleted conditions (Fig. 3B and C). These three regulators shared five genes in their direct regulons: *acnB, ptsG, xylA, lpdA,* and *fumB*. We reasoned that these genes might have a more direct role in regulating iron-mediated motility, and indeed, the *lpdA* mutant resulted in dysregulation of iron-mediated motility at a transcriptional and to a lesser extent, the protein level (Fig. 4B and 5A). There remained a subtle effect of iron-regulated flagellar production (Fig. S5) but at a significantly lower level than observed with WT cells (Fig. 5B and C).

Therefore, we believe that LpdA is one of several factors that can control this response. Given the strong dysregulation at the gene transcription level but lowered amounts at the protein level and even subtler effect at the level of flagellar production, perhaps the other factors are exerting their control at a post-transcriptional stage, an effect that our qRT-PCR-based screen would not detect. A previous study (47) found several small non-coding RNAs (sRNAs) were responsible for regulating motility at the level of *flhDC*. Future studies would be key potentially looking at the protein level or investigating sRNAs encoded within the flagellar machinery that could be controlling this phenomenon in tandem with *lpdA*.

We were surprised that *lpdA* appears to affect iron-mediated motility, given that it has no known iron-binding site. Previous studies have investigated the role that *lpdA* plays in metabolism; it encodes a part of the pyruvate dehydrogenase complex that connects glycolysis with the TCA cycle (36). However, it is worth noting that there are functionally redundant enzymes to LpdA such as PoxB (48) that can compensate for the absence of LpdA, enabling an *lpdA* mutant to utilize glucose as a sole carbon source. Based on its role in metabolism, we hypothesized that *lpdA* would not contribute to pathogenesis since that glycolysis is dispensable for UPEC during UTI (49, 50). However, the mutant had a significant and severe defect in all three organ sites tested (Fig. 6B). To define this loss of fitness more clearly from either metabolic dysfunction or motility, we competed a non-motile Δ*fliC* strain against the *lpdA* mutant, defective in both metabolism and motility (Fig. 6C). Previous studies (39) have shown that a *fliC* mutant is severely attenuated in the mouse model, on the order of 10,000-fold reduced in the kidneys. While the *lpdA* mutant had a defect compared to *fliC*, the magnitude of the loss of fitness was smaller than when *lpdA* was co-challenge against WT. This demonstrates that the *lpdA* defect is largely attributable to the loss of motility rather than metabolic dysfunction.

It was interesting to find that a metabolic gene would so drastically affect flagella-mediated motility, and it is exciting to speculate on the mechanism driving this result. Loss of LpdA results in increased intracellular glucose (35), and it has been established that glucose suppresses production of flagella in non-pathogenic *E. coli* (43). It is possible that the buildup of intracellular glucose levels in the *lpdA* mutant results in the reduction of motility. Furthermore, elevated levels of glucose suppress *crp* expression (51), and since deletion of *crp* resulted in dysregulation of iron-mediated *fliC* transcription, potentially, the intracellular conditions of the *lpdA* mutant partially recapitulate the conditions of the *crp* mutant. Future studies should include the addition of glucose to the same growth conditions to chemically suppress the iron-regulated transcription of *fliC,* thus phenocopying the *lpdA* mutant.

Our study highlights the possible distinctions between uropathogenic bacteria and their commensal counterparts. This work further emphasizes the importance of studying differential regulatory networks in pathogens versus commensals, even among the same bacterial species. We discovered a novel regulatory pathway in UPEC that leads to the upregulation of *fliC* and surface-expressed flagella that is specifically deployed in an iron-depleted environment. This contributes to our biological understanding of the myriad of host virulence mechanisms and how they contribute to disease. Linking motility and iron responsiveness, two highly studied virulence mechanisms, opens new avenues of research for this ubiquitous pathogen, and the findings there can potentially be applied to other pathogenic strains of *E. coli* or other uropathogens.

## MATERIALS AND METHODS

### Bacterial culture conditions, growth curves, mutant construction, and complementation

*E. coli* CFT073 was routinely cultured at 37°C with aeration in LB unless otherwise stated. Mutant and complemented strains were cultured with antibiotics when grown overnight. Mutants were constructed using λ-red mutagenesis, and complementation vectors were constructed with Gibson assembly. Strains used are in Table 1, and primers used are in Table 2. See Text S1 for detailed description.

**TABLE 1:** List of strains and plasmids

Strain or plasmid	Genotype or description	Reference/source
Strains		
CFT073 (WT)	*E. coli* strain isolated from a patient with acute pyelonephritis	(17)
Δ*fis*	CFT073 *fis*::*kan*, Kan^r^	This study
Δ*lrp*	CFT073 *lrp*::*kan*, Kan^r^	This study
Δ*ihf*	CFT073 *ihf*::*kan*, Kan^r^	This study
Δ*crp*	CFT073 *crp*::*kan*, Kan^r^	This study
Δ*arcA*	CFT073 *arcA*::*kan*, Kan^r^	This study
Δ*narL*	CFT073 *narL*::*kan*, Kan^r^	This study
Δ*fnr*	CFT073 *fnr*::*kan*, Kan^r^	This study
Δ*hns*	CFT073 *hns*::*kan*, Kan^r^	This study
Δ*acnB*	CFT073 *acnB*::*kan*, Kan^r^	This study
Δ*fumB*	CFT073 *fumB*::*kan*, Kan^r^	This study
Δ*xylA*	CFT073 *xylA*::*kan*, Kan^r^	This study
Δ*lpdA*	CFT073 *lpdA*::*kan*, Kan^r^	This study
Δ*ptsG*	CFT073 *ptsG*::*kan*, Kan^r^	This study
WT eV	CFT073 expressing empty pGEN vector	This study
Δ*lpdA* eV	Δ*lpdA* expressing empty pGEN vector	This study
Δ*lpdA*^+*lpdA*^	Δ*lpdA* expressing *lpdA* in *trans* under the control of the native promoter	This study
Δ*fliC*	CFT073 *lpdA*::*cam*, Cam^r^	This study

Plasmids		
*fliC lux*	*lux* operon (*luxABCDE*) under control of the CFT073 *fliC* promoter	(12)
Negative *lux*	*lux* operon (*luxABCDE*) without promoter	(12)
pGEN eV	Low copy number, promoterless plasmid, Amp^r^	(12)
pGEN *lpdA*	*lpdA* with native promoter, cloned from CFT073 via Gibson assembly, Amp^r^	This study

**TABLE 2:** Primers used in study^*d*^

Gene or plasmid	Forward primer	Reverse primer
*fis* ^*a*^	TTTTGCGTAAACAGAAATAAAGAGCTGACAGAACTGTGTAGGCTGGAGCTGCTTC	AGCGCCTTTTTAATCAAGCATTTAGCTAACCTGAAATGGGAATTAGCCATGGTCC
*lrp* ^*a*^	TTTCGGTCTATCGTGACGGGTAGCGACTCTGAACAGTGTAGGCTGGAGCTGCTTC	TAATCAAAATACGCCGATTTTGCACCTGTTCCGTGATGGGAATTAGCCATGGTCC
*Ihf* ^*a*^	GCCGCTTAATTTGCCTTTAAGGAACCGGAGGAATCGTGTAGGCTGGAGCTGCTTC	AGGTGCTTTTCTCGCGTTCAAGTTTGAGTAAAAACATGGGAATTAGCCATGGTCC
*crp* ^*a*^	GCTCTGGAGAAAGCTTATAACAGAGGATAACCGCGCGTGTAGGCTGGAGCTGCTTC	GGCGCGCTACCAGGTAACGCGCCACTCTGACGGGAATGGGAATTAGCCATGGTCC
*arcA* ^*a*^	CTGTTTCGATTTAGTTGGCAATTTAGGTAGCAAACGTGTAGGCTGGAGCTGCTTC	AAAAGCGCCGTTTTTATTGACGGTGGTAAAGCCGAATGGGAATTAGCCATGGTCC
*narL* ^*a*^	CTTTCACAGACGTCCAAGGAGATACCCATGAGTAAGTGTAGGCTGGAGCTGCTTC	CATTGTCAAACGACGAACTGCGCTGGGAACCGTAAATGGGAATTAGCCATGGTCC
*fnr* ^*a*^	GGAATTCTCTGCTGTTAAGGTTTGCTTAGACTTACGTGTAGGCTGGAGCTGCTTC	GATATGACAGAAGGATAGTGAGTTATGCGGAAGAAATGGGAATTAGCCATGGTCC
*hns* ^*a*^	ACAAACCACCCCAATATAAGTTTGAGATTACTACAGTGTAGGCTGGAGCTGCTTC	TGGCGGGATTTTTAGCATGTGCAATCTACAAAAGAATGGGAATTAGCCATGGTCC
*acnB* ^*a*^	ATGTTGCTTTTTTGTAAACAGATTAACACATCGTCGTGTAGGCTGGAGCTGCTTC	TTAAACCGCAGTCTGGAAAATCACCCCGTCCGCTTATGGGAATTAGCCATGGTCC
*fumB* ^*a*^	TCGAATAACAAATACAGAGTTACAGGCTGGAAGCTGTGTAGGCTGGAGCTGCTTC	CTGGCAGCATGCTGCCAGGCGCTGGGCCGAAGAGGATGGGAATTAGCCATGGTCC
*xylA* ^*a*^	ACGACATCATCCATCACCCGCGGCATTACCTGATTGTGTAGGCTGGAGCTGCTTC	CTGATAACCGGGCCAACGGACTGCACAGTTAGCCGATGGGAATTAGCCATGGTCC
*lpdA* ^*a*^	CAATTTTGTAAAATACCGACGGATAGAACGACCCGGTGTAGGCTGGAGCTGCTTC	TTCTTAATTTGCCGGATGTTCCGGCAAACGAAAAAATGGGAATTAGCCATGGTCC
*ptsG* ^*a*^	AAAAAAAGCACCCATACTCAGGAGCACTCTCAATTGTGTAGGCTGGAGCTGCTTC	ATCTGGCTGCCTTAGTCTCCCCAACGTCTTACGGAATGGGAATTAGCCATGGTCC
*fliC* ^*a*^	TCAACGACTTGCAATATAGGATAACGAATCGTGTAGGCTGGAGCTGCTTC	CGTCAGTCTCAGTTAATCAGGTTACGGCGAATGGGAATTAGCCATGGTCC
P_native_*lpdA*^*b*^	ATTCCTGCAGGGCATGCCCCTAAAAGAGCCGGCCCAACGG	GTACCAAGCTTCATATGCCCTTACTTCTTCTTCGCTTTCGGGTTCG
pGEN^*b*^	AATTCCTGCAGGGCATGCCCC	GTACCAAGCTTCATATGCCC
*flhD* ^*c*^	GTTAGCGGCACTGACTCTTC	CGTCAACTGAGTAATCGTCTGG
*fliC* ^*c*^	ACAGCCTCTCGCTGATCACTCAAA	GCGCTGTTAATACGCAAGCCAGAA
*fliA* ^*c*^	CCTGCGAGTGTTGAATTGGATG	CTCGAATACGTTGCACAGCATAAG
*cheW* ^*c*^	ACACGCCAGCGTTTATCA	CGTTATAGTCCACATCCACCTG
*cheY* ^*c*^	CGACTGGAACATGCCCAATA	CGCTTCTGCAGTCACCATTA
*gapA* ^*c*^	CGACCTGTTAGACGCTGATTAC	CGATCAGATGACCGTCTTTCAC

^*a*^ Used for λ-red mutagenesis.

^*b*^ Used for Gibson assembly.

^*c*^ Used for qRT-PCR.

^*d*^ Primers are listed in 5′ to 3′; underlined sequences for mutant construction indicate regions homologous to gene of interest.

### *fliC* promoter luminescence activity

We used two previously published reporter constructs (12): one where the CFT073 *fliC* promoter controlled luciferase production (*luxABCDE*) and a promoter-less negative control. We cultured both strains overnight with appropriate antibiotics at 37°C with aeration. The next morning, cultures were back diluted 1:100 into LB, LB supplemented with 300 µM dip (iron deplete), and LB supplemented with 300 µM dip and 300 µM FeCl_3_ (iron replete) and cultured at 37°C with aeration. Cultures were sampled every hour for 6 hours for growth (via OD_600_) and luminescence. Luminescence was quantified with a Synergy H1 reader (Agilent BioTek) using a black-sided 96-well plate. Calculations for normalization are in Text S1.

### Swimming assay

CFT073 motility was assessed in semi-soft swimming agar, formulated as previously described (52). Briefly, after overnight growth, bacterial cultures were normalized to an OD_600_ = 1. Using a sterile inoculating needle, bacteria were introduced to the center of the agar. Plates were incubated for 16 hours at 30°C. Swim diameters were measured and recorded in millimeters.

### Luminescent swimming assay

Overnight cultures were normalized to an OD_600_ = 1. These cultures were inoculated into semi-soft (0.25% agar) LB, iron-depleted, iron-replete agar and incubated for 16 hours at 37°C. Plates were imaged on a Biorad imager using the chemiluminescent channel to visualize *fliC* promoter activity. The bright outer ring of swimming bacteria was measured at its thickest point, and all images used are in Fig. S2.

### Quantitative reverse-transcriptase PCR

qRT-PCR was conducted as previously published (27). Overnight cultures were back diluted 1:100 into LB medium as well as the iron-depleted and iron-replete conditions. Strains were cultured for 5 hours at 37°C with aeration before harvest and treatment with bacterial RNAprotect (Qiagen). Bacterial pellets were stored at −80°C until RNA isolation.

RNA was isolated using Qiagen’s RNAeasy Kit. Genomic DNA was removed with Turbo DNAse-Free Kit, and then RNA was converted into cDNA using Biorad’s iScript. qRT-PCR reactions were performed on 10 ng of cDNA in technical duplicate using SyberGreen reagent and the Quantstudio3 (Applied Biosystems). Gene transcription was calculated using the 2^−ΔΔCT^ method with *gapA* as a housekeeping gene (27) and compared to the LB-only condition. Primers used are in Table 2.

### Western blot

Strains were cultured as described for qRT-PCR. Cultures were normalized to an OD_600_ = 0.4, pelleted at low speed (<5,000 RPM), resuspended in SDS-loading buffer + 2.5% 2-mercaptoethanol (BME), and boiled. Proteins were separated on a 4%–20% SDS-PAGE gel (Biorad) and transferred to nitrocellulose. Membranes were probed with αH1 FliC antibody (Rockland), using a secondary αrabbit-HRP for visualization.

### Electron microscopy

Overnight cultures were back diluted 1:100 into indicated medium and grown for 5 hours. A drop of bacterial culture was incubated on glow-discharged carbon-coated copper grids, 400 mesh (EMS), then fixed with 2.5% glutaraldehyde (EMS), washed with water, and stained with 1% phosotungistic acid. Samples were imaged using a Morgangi (FEI, Hillsboro, OR) operated at an acceleration voltage of 100 kV and equipped with a 1k × 1k charge-coupled device camera (ATM) at 1,800× magnification. See Text S1 for detailed description.

### *Ex vivo* competition in murine organ homogenate

Bacterial strains were grown overnight, normalized, and mixed at a 1:1 ratio to make an OD_600_ = 2.0 input. Pooled human urine was collected and filter sterilized. Murine organs were harvested from naïve, female CBA/J mice and homogenized in 1 mL of sterile PBS. Two microliters of the bacterial input was inoculated into 198 µL of human urine or organ homogenate in a flat bottom 96-well plate and incubated statically at 37°C. Samples were collected at 6, 24, and 48 hpi for drip plating on both plain and antibiotic agar for enumeration of CFU.

### Murine model of ascending UTI

Mice were inoculated as previously published (38). Briefly, female CBA/J mice were transurethrally inoculated with 10^8^ CFU of 1:1 mixed WT and *lpdA* mutant of CFT073, or *lpdA* mutant and *fliC*. After 48 hours, urine, bladder, and kidneys were harvested to enumerate bacterial burden with differential plating on LB agar plates with or without antibiotics to quantify the ratio. This ratio was compared to the ratio of the input to determine a competitive index (27).

## SUPPLEMENTAL MATERIAL

Text S1 mbio.01048-24-s0001.docx Supplemental methods.

Fig. S1 mbio.01048-24-s0002.tif Growth of *E. coli* CFT073 in LB medium supplemented with increasing concentrations of dip.

Fig. S2 mbio.01048-24-s0003.tif Measuring leading edge of swimming colonies expressing *fliC* promoter reporter construct.

Fig. S3 mbio.01048-24-s0004.tif Growth of WT and mutant *E. coli* in LB, iron-depleted, and iron-replete conditions.

Fig. S4 mbio.01048-24-s0005.tif Growth of WT and mutant *E. coli* in LB, iron-depleted, and iron-replete conditions

Fig. S5 mbio.01048-24-s0006.tif Flagella per bacterial cell in a *lpdA* mutant.

Fig. S6 mbio.01048-24-s0007.tif Complementation of the *lpdA* mutant.

Fig. S7 mbio.01048-24-s0008.tif Comparison of 48-h co-challenge between WT and *lpdA* in *ex vivo* and *in vivo* conditions.

Table S1 mbio.01048-24-s0009.xlsx Upregulated genes.

ASM does not own the copyrights to Supplemental Material that may be linked to, or accessed through, an article. The authors have granted ASM a non-exclusive, world-wide license to publish the Supplemental Material files. Please contact the corresponding author directly for reuse.
