# Supplementary material for: Iron limitation induces motility in uropathogenic E. coli CFT073 partially through action of LpdA
Source: mBio. 2024 Jun 14;15(7):e01048-24. doi: 10.1128/mbio.01048-24 (PMC11253704; doi:10.1128/mbio.01048-24)
Supplement: Text S1 — Supplemental methods. [file mbio.01048-24-s0001.docx]

**Supplemental Text 1**

**Bacterial growth and culture**. E. coli CFT073 was routinely cultured overnight in Luria-Bertani broth (LB, 10 g tryptone, 5 g yeast extract, 0.5 g NaCl) with aeration at 37oC, unless otherwise stated. Cultures were inoculated with a single isolated colony. Kanamycin (25 µg/mL) or chloramphenicol (25 µg/mL) was added to the medium for designated mutant constructs, and ampicillin (100 ug/mL) was added for maintenance of plasmids. Strains used are in Table 1.

**Growth curves**. Strains were cultured overnight in LB with appropriate antibiotics at 37°C with aeration. The next day, cultures were back-diluted 1:100 into indicated conditions and incubated with shaking at 37°C for 16 hours using the Bioscreen-C automatic growth curve analyzer.

**Bacterial mutants and genetic complementation.** The lambda red recombinase system was used to generate mutants in CFT073 (52). Homology regions of 35 bp, both upstream and downstream of the gene of interest, were used to flank the kanamycin resistance cassette amplified from pKD4, or chloramphenicol cassette from pkD3. This PCR product was introduced into wild-type strain carrying the recombinase expression system encoded on pKD46 (52). Complementation vectors in pGEN were constructed using Gibson Assembly. The native promoters were identified by taking sequence upstream of lpdA until the next gene was reached, resulting in a 144 bp stretch. Primers used are in Table 2.

***fliC* promoter luminescence activity.** We used two reporter constructs that have been previously published (12)). One, where the CFT073 *fliC* promoter-controlled luciferase production (*luxABCDE*), and a promoter-less negative control. We cultured both strains overnight with appropriate antibiotics at 37°C with aeration. The next morning, cultures were back-diluted 1:100 into LB, LB supplemented with 300 μM dip (iron-deplete), and LB supplemented with 300 μM dip and 300 μM FeCl_3_ (iron-replete) and cultured at 37°C with aeration. Cultures were sampled every hour for six hours for growth (via OD_600_) and luminescence. Luminescence was quantified with a Synergy H1 reader (Agilent BioTek) using a black-sided 96-well plate. Luminescence was normalized by colony forming units (CFU) at an OD_600_ reading of 1=7.5x10^8^ CFU/mL, the background luminescence calculated by the values of the negative control and subtracted from the experimental group. Finally, these normalized luminescence values were reported per 10^6^ CFU.

**Electron microscopy.** Overnight cultures were back-diluted 1:100 into indicated medium and grown for five hours. A drop of bacterial culture was incubated on glow-discharged carbon coated copper grids, 400 mesh (EMS) for three minutes. Excess culture was wicked away and bacteria fixed on grids with 2.5% glutaraldehyde (EMS) for five minutes. Grids were then washed by being plunged in water ten times and the excess water wicked away. A 1% phosotungistic acid stain was applied to the grids for 30 seconds to stain the samples and the excess aspirated away to dry the grids. Samples were imaged using a Morgangi (FEI, Hillsboro, OR) operated at an acceleration voltage of 100 kV and equipped with a 1kx1k charge-coupled-device (CCD) camera (ATM) at 1,800x magnification.
