# Supplementary figures and images for: Iron limitation induces motility in uropathogenic E. coli CFT073 partially through action of LpdA
Source: mBio. 2024 Jun 14;15(7):e01048-24. doi: 10.1128/mbio.01048-24 (PMC11253704; doi:10.1128/mbio.01048-24)

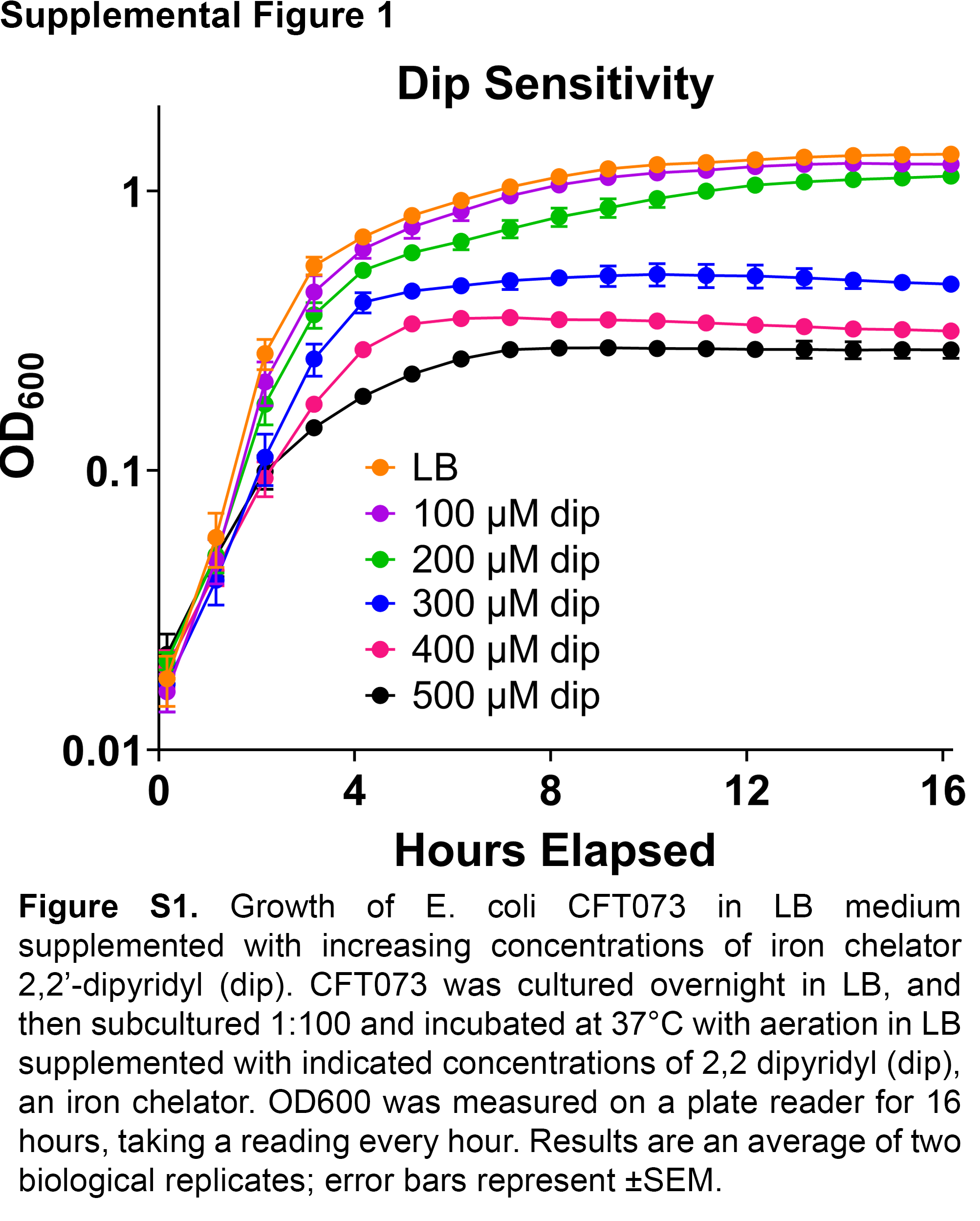

Supplement: Fig. S1 — Growth of E. coli CFT073 in LB medium supplemented with increasing concentrations of dip. [file mbio.01048-24-s0002.tif]

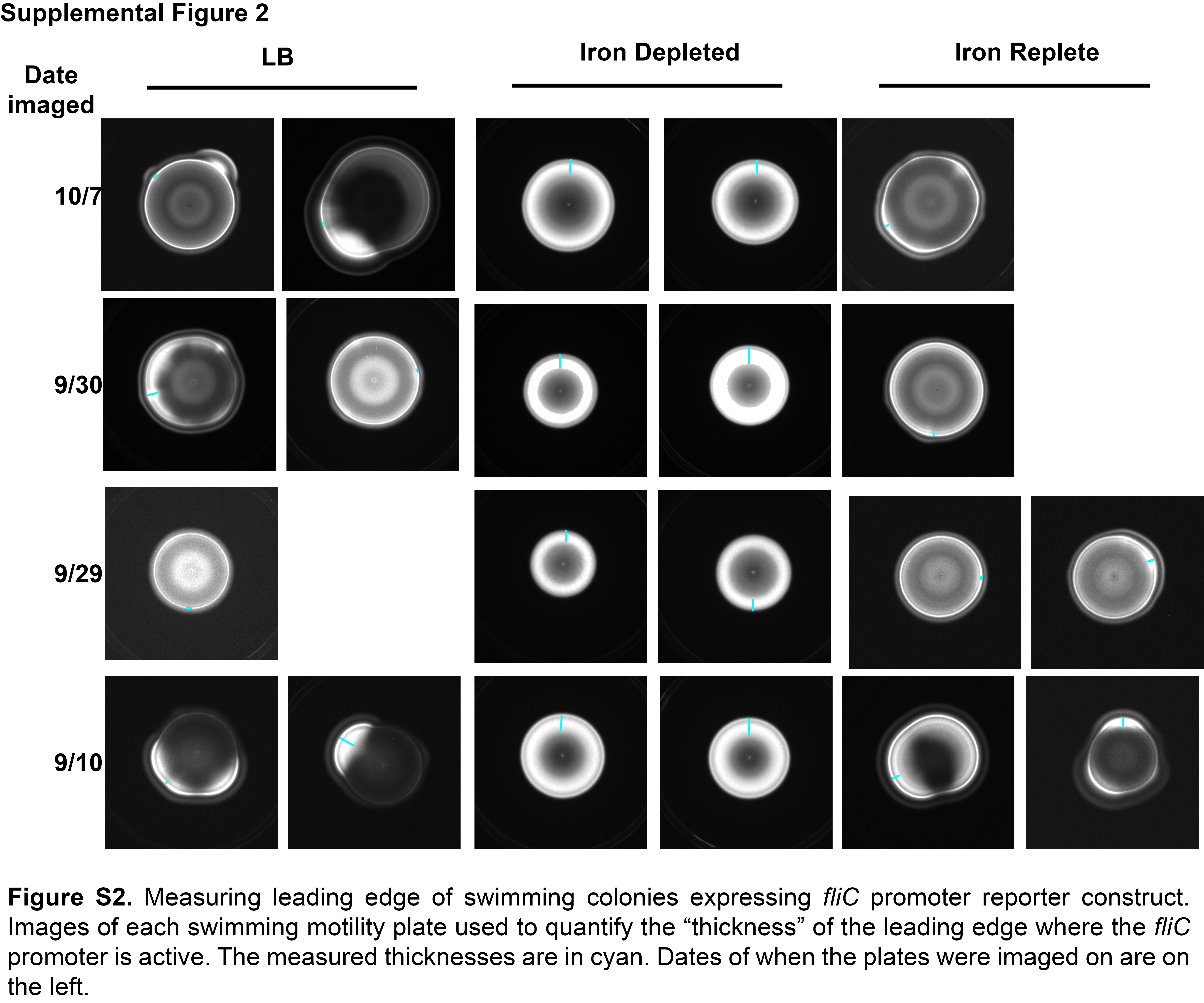

Supplement: Fig. S2 — Measuring leading edge of swimming colonies expressing fliC promoter reporter construct. [file mbio.01048-24-s0003.tif]

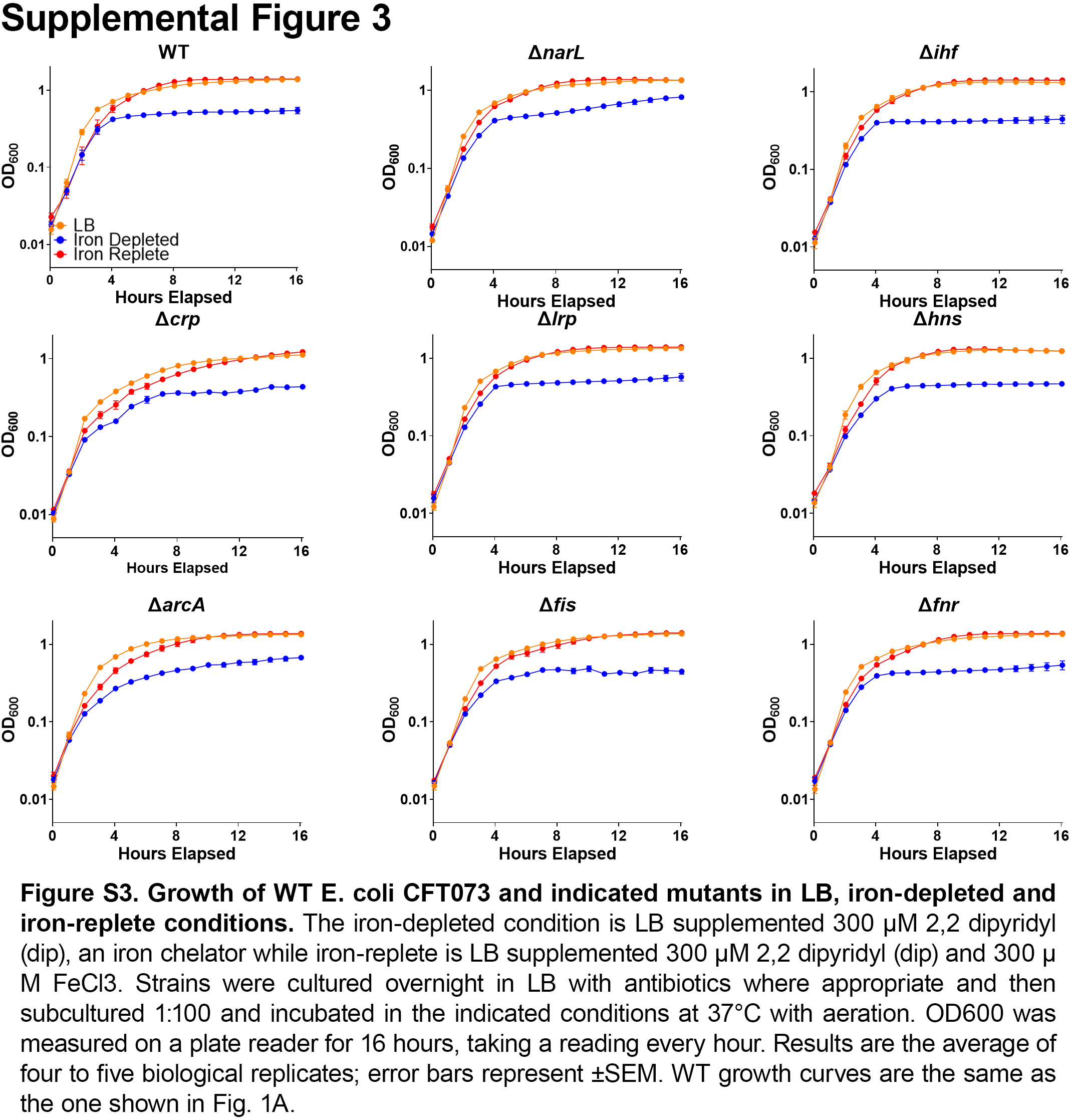

Supplement: Fig. S3 — Growth of WT and mutant E. coli in LB, iron-depleted, and iron-replete conditions. [file mbio.01048-24-s0004.tif]

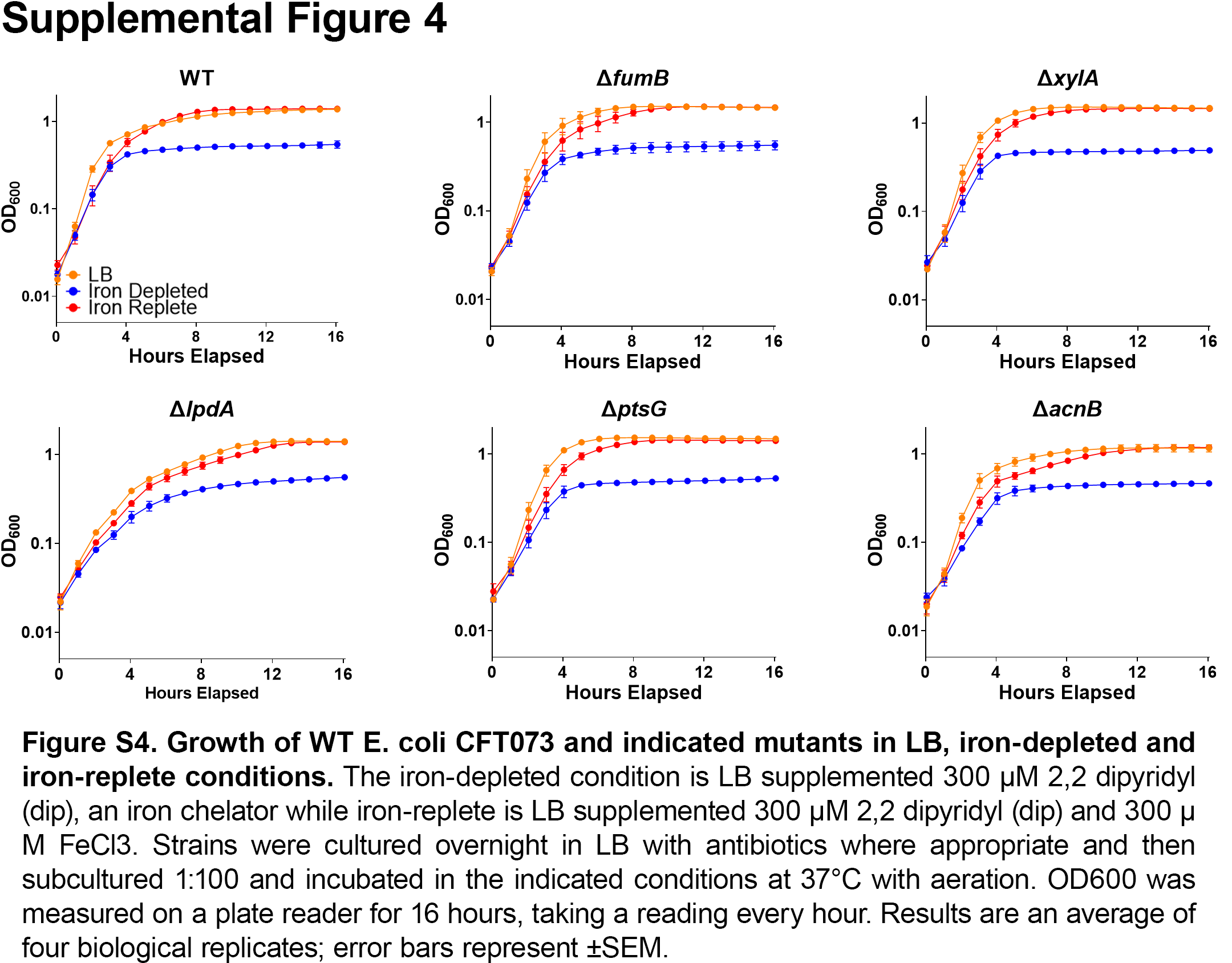

Supplement: Fig. S4 — Growth of WT and mutant E. coli in LB, iron-depleted, and iron-replete conditions [file mbio.01048-24-s0005.tif]

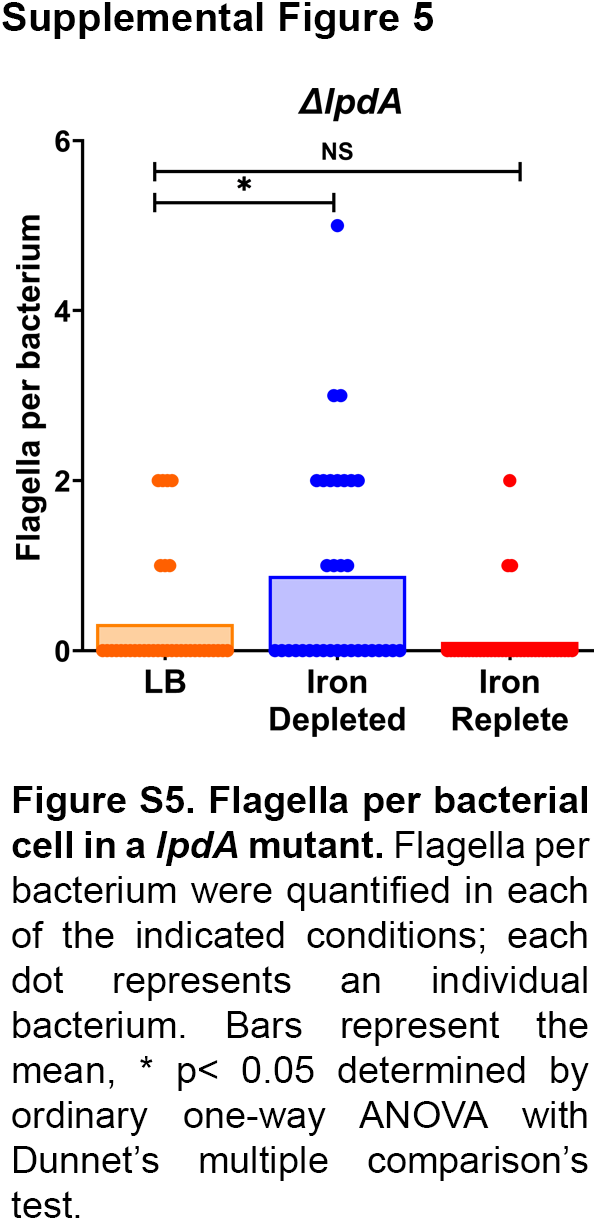

Supplement: Fig. S5 — Flagella per bacterial cell in a lpdA mutant. [file mbio.01048-24-s0006.tif]

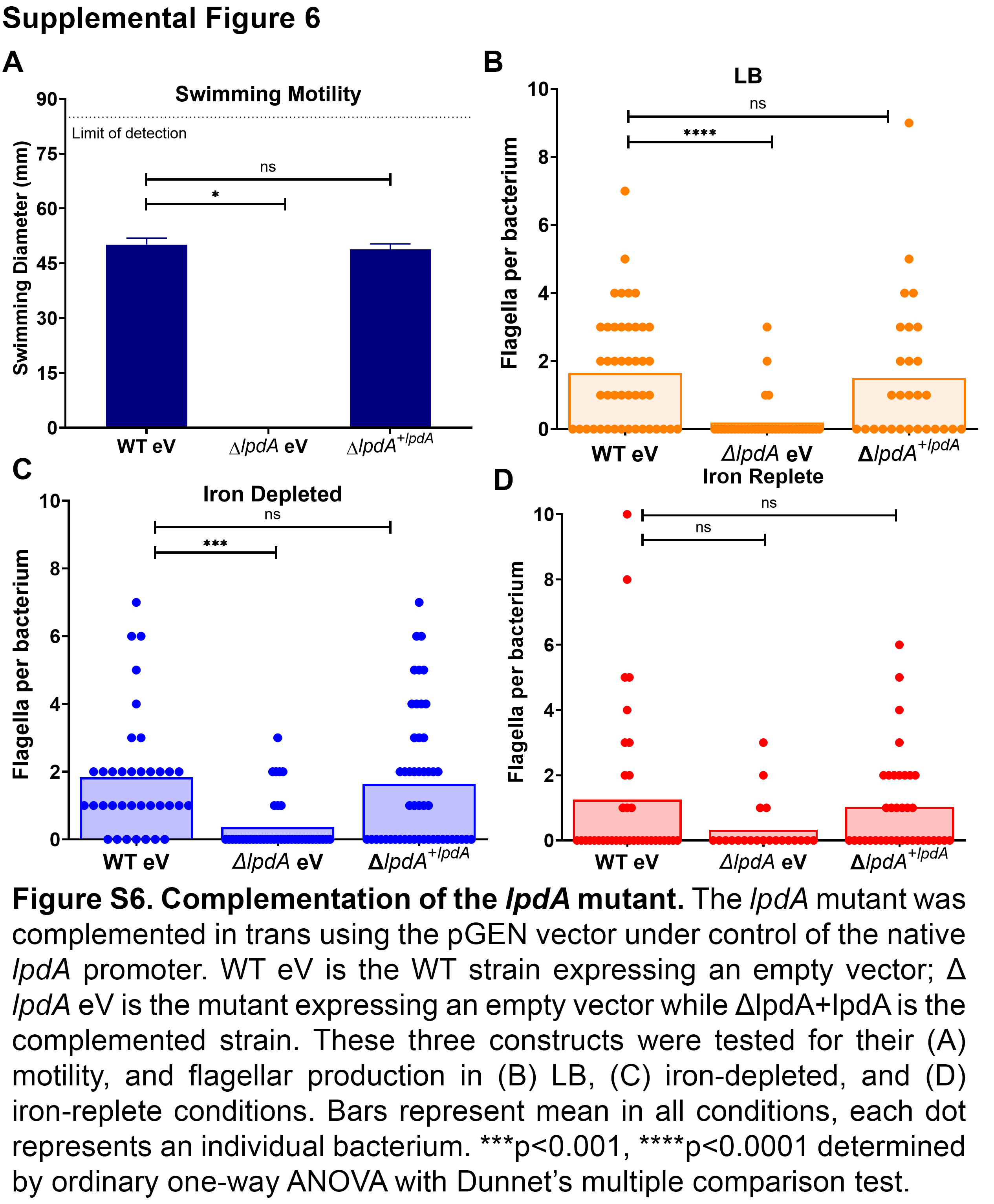

Supplement: Fig. S6 — Complementation of the lpdA mutant. [file mbio.01048-24-s0007.tif]

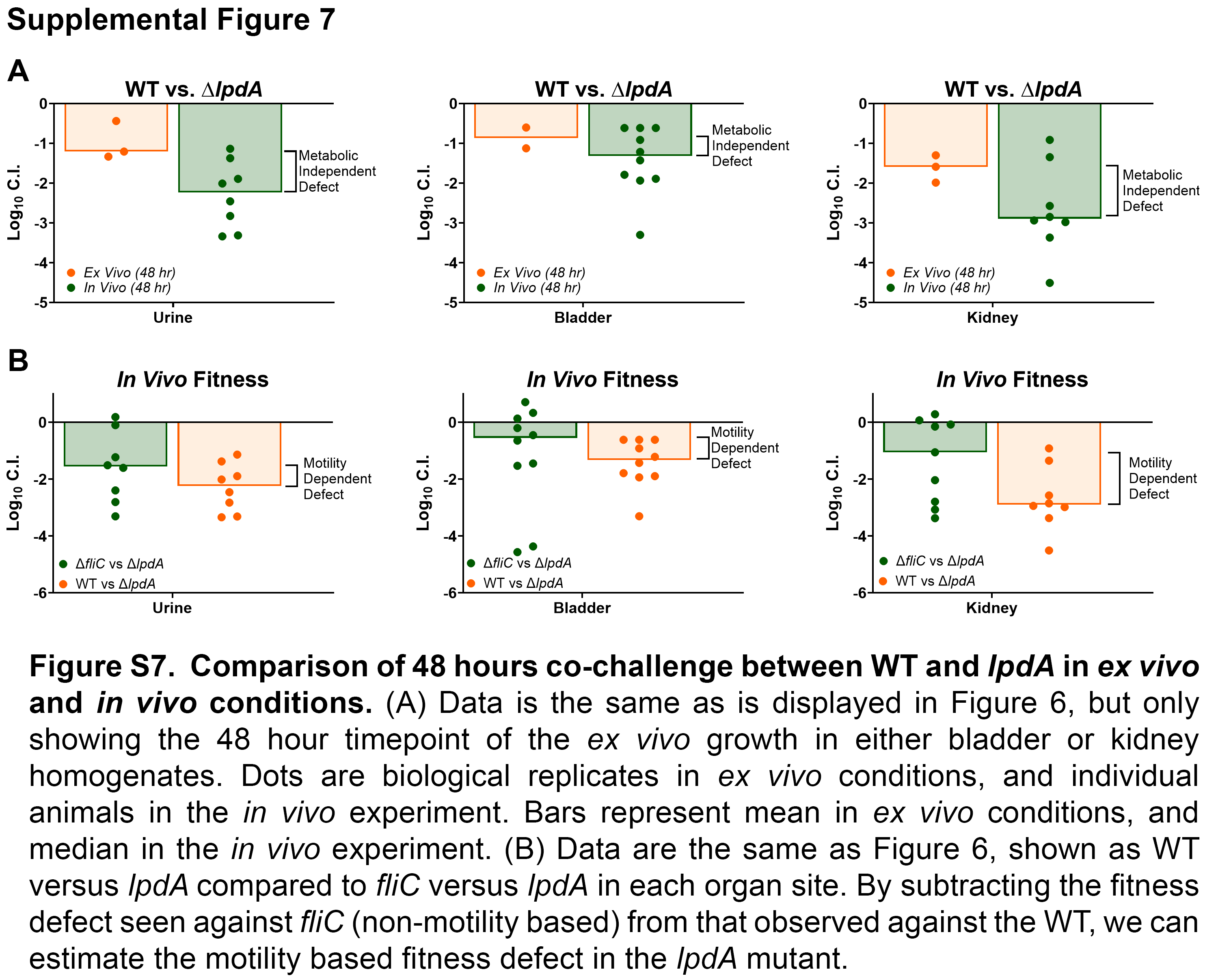

Supplement: Fig. S7 — Comparison of 48-h co-challenge between WT and lpdA in ex vivo and in vivo conditions. [file mbio.01048-24-s0008.tif]
